# Effect of *Candida albicans* Suspension on the Mechanical Properties of Denture Base Acrylic Resin

**DOI:** 10.3390/ma15113841

**Published:** 2022-05-27

**Authors:** Grzegorz Chladek, Michał Nowak, Wojciech Pakieła, Anna Mertas

**Affiliations:** 1 Chair of Engineering Materials and Biomaterials, Faculty of Mechanical Engineering, Silesian University of Technology, 18a Konarskiego Str., 41-100 Gliwice, Poland; wojciech.pakiela@polsl.pl; 2 Nova Clinic, 22 Jankego Str., 40-612 Katowice, Poland; michalnowak83@yahoo.pl; 3 Department of Microbiology and Immunology, Faculty of Medical Sciences in Zabrze, Medical University of Silesia in Katowice, 19 Jordana Str., 41-808 Zabrze, Poland; amertas@sum.edu.pl

**Keywords:** *Candida albicans*, dentures, mechanical properties, colonization, penetration, polymethyl methacrylate

## Abstract

Yeast-like fungi such as *Candida albicans* (*C. albicans*) are the primary pathogenic microorganism in the oral cavity of denture wearers. The research available so far, conducted according to a protocol based on the exposure of specimens to a *C. albicans* suspension and their cutting with water cooling, shows that hard polymethyl methacrylate (PMMA) prosthetic materials are not only surface colonized, but also penetrated by microorganisms in a short time. This justifies the hypothesis that exposure to a suspension of the *C. albicans* strain causes the changes in mechanical properties due to surface colonization and/or penetration of the samples. In the current study, the chosen mechanical properties (flexural strength, flexural modulus, tensile strength, impact strength, ball indentation hardness, and surface Vickers hardness at 300 g load) of the PMMA denture base material Vertex RS (Vertex-Dental, The Netherlands) exposed for 30, 60, and 90 days to a suspension of *C. albicans* were investigated. The potential penetration of yeast was examined on the fractured surfaces (interior of specimens) to eliminate the risk of the contamination of samples during cutting. There was no influence on the flexural strength, flexural modulus, tensile strength, impact strength, or ball indentation hardness, but a significant decrease in surface hardness was registered. Microscopic observations did not confirm the penetration of *C. albicans*. On the surface, blastospores and pseudohyphae were observed in crystallized structures and in traces after grinding, which indicates that in clinical conditions, it is not penetration but the deterioration of surface quality, which may lead to the formation of microareas that are difficult to disinfect, causing rapid recolonization.

## 1. Introduction

The functioning of polymethyl methacrylate (PMMA) prosthetic materials in the context of the presence of pathogenic microorganisms in the oral cavity, especially *Candida* yeast strains, is one of the most serious clinical problems. It concerns both the colonization of the tissues under the denture and the properties of the dental materials. The relationship between the pathogenic microflora of the oral cavity and the appearance of stomatitis is well-known [1,2,3], however, this problem has a much wider context, since the relationship between oral microorganisms and diseases of the lungs, cardiovascular system (including heart), kidneys, and the digestive system has been proven [4,5]. Pathogenic microorganism colonized prostheses are believed to adversely affect the general health of treated patients [6]. This is facilitated by the fact that the microorganisms present in the dentures are both aspirated and swallowed with saliva or chewed food. 

It should be emphasized that maintaining the hygiene of dentures and mucosa is a challenge due to the conditions in the oral cavity with 100% humidity, a reduced pH value, increased temperature, no possibility of self-cleaning of the mucosa under the prosthesis by saliva, reduced access of oxygen, and a significant probability of mucosa injury due to hard denture plates [2,7]. Consequently, yeast-like fungi, especially *Candida albicans* (*C. albicans*), which are a part of the commensal oral microflora in many healthy individuals, for denture wearers, can rapidly become pathogenic and are isolated much more frequently, at least in 50% of patients [1,8], but when stomatitis occurs, the percentage exceeds 90% [9,10]. As a result, the research on denture base materials in terms of their colonization by microorganisms and finding effective methods to counteract this colonization are among the most important in the area. Despite many attempts to provide materials with antifungal properties and the use of cleaning agents, this problem has not been solved due to its negative influence on the mechanical and/or aesthetic properties of the materials [7,11,12,13,14,15,16,17], and the limited antimicrobial effectiveness of cleaning agents [18] associated with rapid recolonization (several minutes) after cleaning [19].

Many studies suggest that not only is the surface of the material colonized, but *C. albicans* can penetrate the commonly used acrylic resin [20]. Bulad’a et al. [21] found it inside hard acrylic samples cut with a microtome of *C. albicans* blastopores. Krishnamurthy et al. [22] obtained similar results with a comparable methodology, however, blastopores and hyphae were found inside the material. These studies indicate the probability that the colonization and penetration of materials by *C. albicans* may contribute, especially in the long-term, to the gradual loss of mechanical properties. This seems particularly probable if we take into account that in the above-mentioned studies, the number of cells present inside the samples was counted in the thousands on each of the analyzed planes, and the presence of *C. albicans* inside the materials was demonstrated after a short time [23]. However, this problem has not been investigated thus far. The purpose of the present work was to investigate the influence of exposure to *C. albicans* suspension on the mechanical properties of the polymethyl methacrylate denture base material and to verify previous reports on the penetration of *C. albicans* into the material. Our hypothesis was that exposure to a suspension of *C. albicans* strain causes changes in the mechanical properties due to surface colonization and/or penetration of the samples.

## 2. Materials and Methods

### 2.1. Materials and Sample Preparation

#### 2.1.1. Sample Preparation

A transparent (color no. 4) heat-cured acrylic denture base resin Vertex Rapid Simplified (Vertex-Dental, Soesterberg, The Netherlands) was used and all samples were prepared with the standard flasking technique used in prosthetic dentistry, similar to the one described in a previous work [7]. First, the molds were prepared in dental flasks. The wax models of the sample types were flooded with dental stone (type IV gypsum, Zhermack, Badia Polesine, Italy) to prepare the first part of the mold. After the gypsum set, the mold was placed in the first part of the flask and mounted using model plaster (Stodent II, Zhermack, Badia Polesine, Italy). After setting the model, the plaster separation medium (Divosep, Vertex Dental, Soesterberg, The Netherlands) was applied with a brush. When its setting was finished, the second part of the flask was mounted, dental stone was poured to about 1/4 of its volume; after it set, the flask was filled with model plaster and finally closed with a cover. After model plaster setting, the wax models were removed by smelting and the surfaces of both parts of the mold were covered with separate media (Divosep, Vertex Dental, Soesterberg, The Netherlands). The samples were polymerized according to the manufacturer’s instructions.

After the whole process, the samples were taken out of the mold and their quality was controlled: if bubbles, discontinuities, or other defects occurred, the sample was discarded. The excess material was cut with a scalpel and the samples were individually wet ground for standardization (Labo-Pol25, Struers, Willich, Germany) with P500-grit abrasive paper (tensile strength, ball hardness, impact strength, and flexural strength) or for surface hardness tests with P500, P800, P1200-grit (Struers, Willich, Germany) and finely with 6 µm diamond paste (Struers, Willich, Germany). After the finishing process, all samples were thoroughly rinsed in distilled water and conditioned in distilled water at 37 ± 1 °C for 48 h to remove most of the residual monomer [24,25].

#### 2.1.2. Incubation in the Suspension of *Candida albicans*

Plasma sterilized samples were incubated for 30, 60, and 90 days in Sabouraud liquid medium (bioMérieux, Craponne, France) diluted five times with PBS (control samples—CO) at 37 °C and in a suspension of the reference strain *C. albicans* ATCC 10231 in liquid Sabouraud medium diluted 5-flod with a 0.9% NaCl solution, the final density of the strain suspension was 3 × 10^6^ CFU/mL (test samples—CA) at 37 °C. The control medium and the *C. albicans* suspension in the medium were changed twice a week.

After the end of each of the incubation times, the samples were removed with tweezers and lightly rinsed with a 4% glutaraldehyde solution in 0.9% NaCl solution, and then placed in the solution as above for 2 h. After this time, the samples were tested.

### 2.2. Methods

#### 2.2.1. Tensile Strength Test

For dumbbell-shaped tensile strength tests, samples of type 5B specified by EN ISO 527-2 and 1.5-mm thick specimens were mounted with polymeric tweezers in the jaws of a universal testing machine (Zwick Z020, Zwick GmbH & Com, Ulm, Germany). For each of the experiment conditions, 10 samples were tested. The speed was 5 mm/min [26]. The tensile strength was calculated according to the following equation:

(1) $$T_{s}=\frac{F_{max}}{A}$$

where T_s_ is the ultimate tensile strength (MPa); F*_max_* is the force at rupture (N); A is the initial cross-sectional area (mm^2^).

After testing, the fractured samples were carefully removed in a Petri dish with polymeric tweezers (they were caught near the place of attachment in the jaw) and the parts were placed with a distance that prevents their contact.

#### 2.2.2. Flexural Properties

A three-point bending test based on the ISO 20795-1:2013-07 standard [27] with differences resulting from the aim of the experiment (specimens were tested after exposition). For each of the experimental conditions, 10 specimens measuring 65 mm × 10 mm × 3.3 mm were prepared. The distance between the supports was 50 mm, and the crosshead speed was 5 mm/min. The flexural strength and the flexural modulus were calculated according to the equations.

(2) $$FS=\frac{3Fl}{2bh^{2}}$$

(3) $$E=\frac{F_{1}l^{3}}{4bh^{3}d}$$

where *FS* is the flexural strength (MPa); *E* is the flexural modulus (GPa); *l* is the distance between the supports (mm); *b* and *h* are the width and height (mm); *F* is the maximal force (N); *F*_1_ is the load at a chosen point at the elastic region of the stress–strain plot (kN); and *d* is the deflection at *F*_1_ (mm).

During the test, the samples were secured at their ends, protruding over the supports in such a way that they did not fall down at the moment of fracture, and after the test, they were carefully placed on Petri dishes at a distance that prevented contact. If for any reason a broken specimen fell, or another risk of the contamination of the fractured surface occurred, it was removed from further tests.

#### 2.2.3. Charpy Impact Strength

The Charpy impact strength test was based on the ISO 179-1:2010 standard [28] with differences resulting from the aim of the experiment. For each experimental conditions, 10 unnotched samples measuring 50 mm × 6 mm × 4 mm after incubation were placed horizontally on supports (the distance between them was 40 mm) and the test was performed on a pendulum impact tester (HIT 25P, Zwick GmbH & Com, Ulm, Germany) and a pendulum with an energy of 1 J was used. The impact strength was calculated according to the following equation:

(4) $$a_{cU}=\frac{E}{b\times d}\times 10^{3}$$

where *a_cU_* is the impact strength; *E* is the energy absorbed by breaking the test specimen (J); *b* and *d* are the width and thickness of the specimen, respectively (mm).

#### 2.2.4. Surface Vickers Hardness

For the tests, samples were made with dimensions of 10 × 10 mm and a thickness of 3.0 mm. All samples were made during the same polymerization series to ensure that the results were not affected by this process. Hardness was measured using a Future-Tech FM-700 microhardness tester (Future-Tech Corp, Tokyo, Japan) at a load of 300 g and loading time of 15 s five times for each sample at randomly selected locations with a minimum distance of 2 mm between indentations, and the means of individual specimens were averaged [16,29] on 15 samples per group. The Vickers hardness was calculated automatically by a hardness tester based on the average length of the diagonal left by the indenter.

#### 2.2.5. Ball Hardness

For the tests, samples were made with dimensions of 30 × 40 mm and a thickness of 5.0 mm. All samples were produced during the same polymerization series to ensure that the results were not affected by this process. The ball indentation hardness (*H*) was determined according to the ISO 2039-1 standard [30] on a Zwick 3106 hardness tester (Zwick GmbH & Com, Ulm, Germany) for samples after a particular incubation time. During the test, a steel ball with a diameter of 5 mm was indented into the material under a load of 358 N for 30 s. During the measurements, the indentation of the depth of the ball was measured, and the ball hardness (H) using the surface of the impression was automatically calculated in N/mm^2^. Three samples were made for each condition, and five measurements were made in each of them at randomly selected places with a minimum distance of 10 mm between indentations to calculate the mean hardness value.

#### 2.2.6. Microscopic Evaluation—Colonization and Penetration Evaluation

Scanning electron microscopy (SEM) with a Zeiss SUPRA 35 (Zeiss, Oberkochen, Germany) and an OLYMPUS IX 51 (Olympus, Tokyo, Japan) inverted fluorescence microscope was used for qualitative evaluation to confirm the presence of *C. albicans* on the surfaces and inside the specimens (the potential penetration was evaluated on fractured surfaces—interior of specimens). During the SEM investigations, we randomly chose five halves of the fractured specimens after the tensile strength and flexural strength tests were investigated (a total of 10 halves of the specimens for each exposition condition). Observations were performed at accelerating voltages of 15 kV, and all samples were gold sputtered [7]. The samples (five for each condition) for fluorescence microscopy were carefully rinsed in PBS, then 1–2 drops of Calcofluor White Stain (Sigma-Aldrich, St. Louis, MO, USA) were placed on the microscopic glass, the investigated surface was placed in it, and after 1–2 min of incubation, it was observed at room temperature under UV light using an inverted fluorescence microscope. Fungal organisms appear fluorescent bright green to blue because the Calcofluor White Stain is a non-specific fluorochrome that binds with the cellulose and chitin contained in the cell walls of fungi and other organisms.

#### 2.2.7. Statistical Analysis

Statistical analysis of the results was conducted using the PQStat ver. Software 1.6.6.204 (PQStat Software, Poznań, Poland). The results of the mechanical properties tests were carried out with one-way ANOVA with F* correction (Brown–Forsythe) when the assumption of the equality of variances was not met (α = 0.05). The distributions of the residuals were tested with the Shapiro–Wilk test, and the equality of variances was tested with the Levene test (α = 0.05). Tukey HSD post hoc tests were used (α = 0.05).

## 3. Results

### 3.1. Mechanical Properties

**Figure 1:**
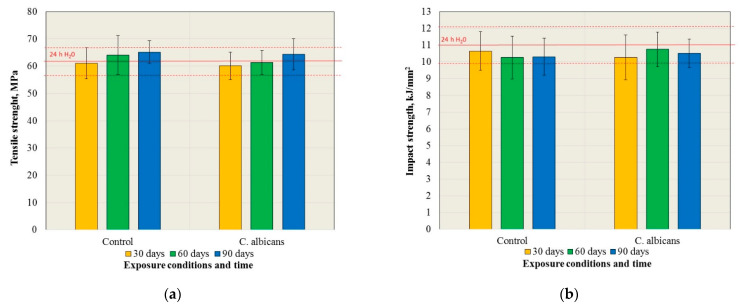
The mean values of the tensile strength (**a**) and impact strength (**b**) with the standard deviations after exposure to the control medium and the *C. albicans* suspension.

The results of the tensile strength tests are presented in Figure 1a. There were no statistically significant changes in tensile strength after exposure to the control medium and the *C. albicans* suspension, but also in comparison to the baseline (24 h H_2_O) (*p* = 0.3245 and *p* = 0.2949, respectively). Furthermore, the tensile strength values did not differ in their statistical significance (*p* > 0.05) for particular times after exposure to the control medium and the suspension of *C. albicans.*

The results of the impact strength tests are presented in Figure 1b. There were no statistically significant changes in the impact strength after exposure to the control medium and the suspension of *C. albicans*, but also in comparison to the 24 h H_2_O (*p* = 0.6457 and *p* = 0.6233, respectively). Furthermore, the impact strength values did not differ in their statistical significance (*p* > 0.05) for the exposure times after exposure to the control medium and the *C. albicans* suspension. 

**Figure 2:**
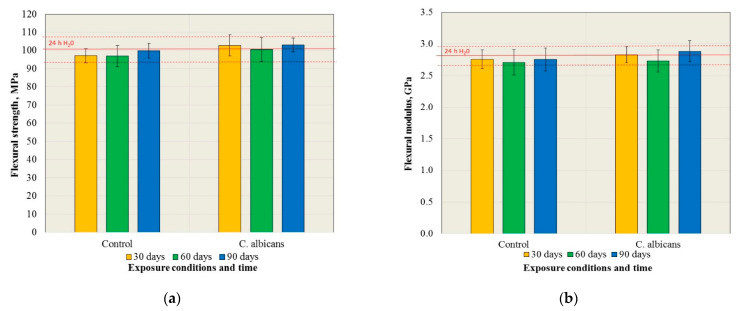
The values of the mean flexural strength (**a**) and flexural modulus (**b**) with the standard deviations after exposure to the control medium and the *C. albicans* suspension.

The results of the flexural strength tests are presented in Figure 2a. There were no statistically significant changes in flexural strength after exposure to the control medium and the *C. albicans* suspension, but also in comparison to the baseline (24 h H_2_O) (*p* = 0.607 and *p* = 0.7516, respectively). Furthermore, the flexural strength values did not differ in their statistical significance (*p* > 0.05) for particular exposure times after exposure to the control medium and the *C. albicans* suspension. The results of the flexural modulus are presented in Figure 2b. There were no statistically significant changes in the flexural strength after exposure to the control medium and to the *C. albicans* suspension, but also in comparison to the baseline (24 h H_2_O) (*p* = 0.6146 and *p* = 0.1848, respectively). Furthermore, the values of the flexural modulus did not differ in their statistical significance (*p* > 0.05) for individual exposure times after exposure to the control medium and the *C. albicans* suspension. 

**Figure 3:**
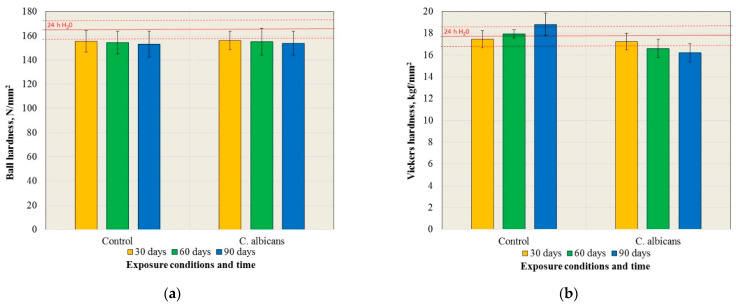
The mean ball hardness (**a**) and Vickers hardness (**b**) values with the standard deviations after exposure to the control medium and the *C. albicans* suspension.

The results of the ball indentation hardness tests are presented in Figure 3a. There were statistically significant changes in the hardness after exposure to the control medium (*p* = 0.0033) and the suspension of *C. albicans* (*p* = 0.0006), however, the results of the post hoc test (Table 1) showed that statistically significant differences were observed only in comparison to the initial hardness value (24 h H_2_O). The ball hardness values did not differ in their statistical significance for individual exposure times after exposure to the control medium and the *C. albicans* suspension.

The results of the Vickers hardness tests are presented in Figure 3b. There were statistically significant changes in the hardness after exposure to the control medium (*p* = 0.0001) and the *C. albicans* suspension (*p* < 0.0001). The results of the post hoc test (Table 2) showed that statistically significant differences were observed for the control conditions after 90 days of exposure compared to the results after all other periods including the starting point (increase in hardness). After exposure to the *C. albicans* suspension, successive reduction (also in comparison to starting point) in the hardness (statistically significant differences) with the prolonging of time was registered. The Vickers surface hardness values after exposure to the control medium and the *C. albicans* suspension did not differ in their statistical significance only after 30 days of exposure, but after 60 and 90 days, the differences for the individual exposure times were statistically significant.

### 3.2. Microscopic Evaluation of Surfaces and Fractures

**Figure 4:**
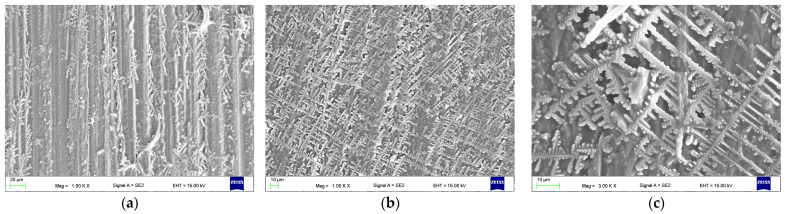
The SEM microphotographs presenting the surface morphologies after 30 days of exposure (**a**) and 90 days of exposure in the control medium (**b**,**c**).

Representative SEM microphotographs that show the surfaces of the control samples are presented in Figure 4. Parallel traces resulting from the grinding of samples with abrasive paper were covered largely with structures related to crystallization in the control medium. With increasing exposure time, a tendency to increase the accumulation of crystalized structures was observed, however, even after 90 days, there were still some microareas where they were hardly present.

**Figure 5:**
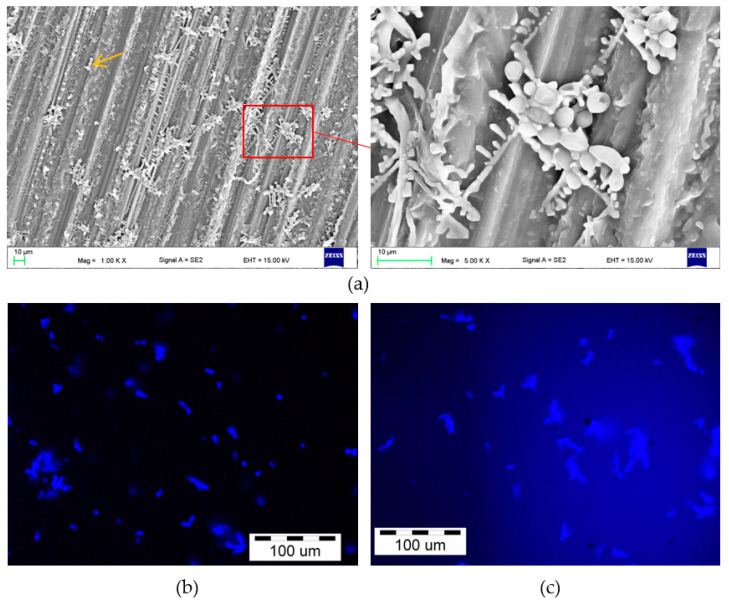
Representative microphotographs of the surface of the samples after 30 days of incubation in the *C. albicans* suspension obtained by SEM (**a**) and fluorescence microscopy (**b**,**c**), yellow arrow—exemplary colony inside the scratch.

**Figure 6:**
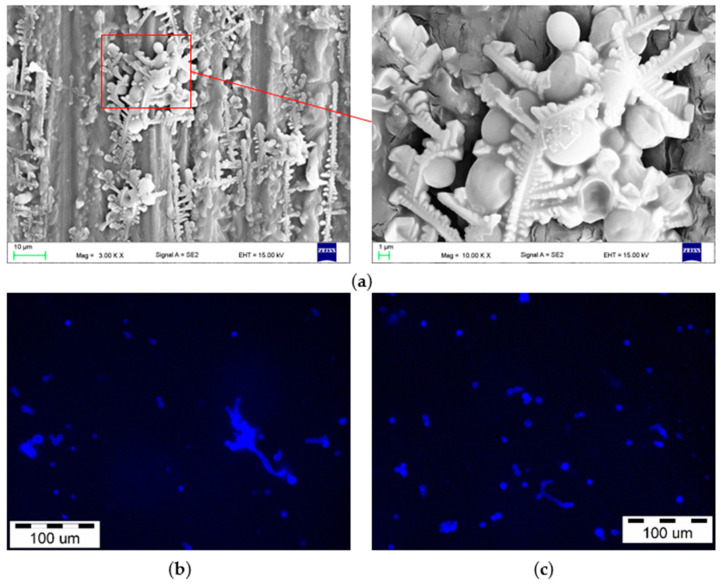
Representative microphotographs of the surface of the samples after 90 days of incubation in the *C. albicans* suspension obtained by SEM (**a**) and fluorescence microscopy (**b**,**c**).

After incubation in the *C. albicans* suspension, the presence of a microorganism on the surface of the samples was confirmed by SEM and fluorescence microscope observations (Figure 5 and Figure 6). Numerous blastospores as well as pseudohyphae were observed after all incubation times, but the hyphae form was found episodically only after 90 days. Colonies composed of blastospores were the most common, differing in their number and also containing pseudohyphae. There was no obvious trend showing an increase in the number of cells in individual colonies related to an increase in the incubation time. This number usually ranged from a few to over a dozen, and only sporadically more, but there were no colonies characterized by cell numbers visibly exceeding the ones presented in Figure 5 and Figure 6. The SEM observations showed that colonies were formed in crystallized structures and much less frequently inside the scratches after grinding (e.g., Figure 5a, yellow arrow).

For both the control and the samples exposed to *C. albicans*, no signs of surface deterioration (e.g., cracks) were observed. 

**Figure 7:**
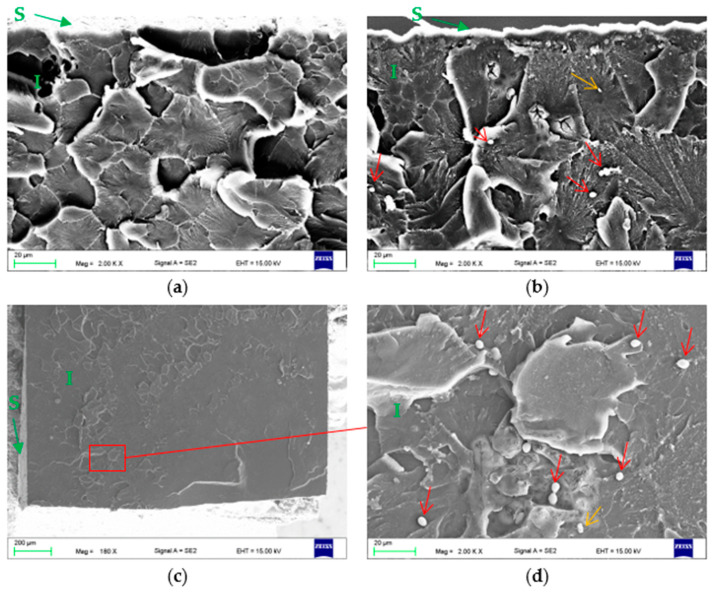
Representative SEM microphotographs of the fractured surfaces (interior of specimens) of the samples after 60 days (**a**,**b**) and 90 days (**c**,**d**) incubation in *C. albicans* suspension. S—surface, I—interior of specimen, red arrows—*C. albicans* blastospores, yellow arrows—contaminations.

In Figure 7, exemplar fractures of the PMMA samples after flexural strength tests are shown. They were examined to assess the potential penetration of *C. albicans*. All fractures (after exposure to the control medium and the *C. albicans* suspension) were characterized by a similar morphology. Typical areas representing the brittle fracture mode (compact and smooth surface fields) and an intermediate (brittle to ductile) fracture mode (a jagged and rough appearance) were observed. Cells of *C. albicans* were not found in any of the fractures after 30 days of exposure. After 60 days, one of the fractures analyzed showed the presence of three single blastospores and one colony consisting of two cells (Figure 7b) at a distance of approximately 60–100 µm from the sample surface. In another fracture, two single blastospores were found approximately 200 µm from the sample surface. No blastospores or other forms of *C. albicans* were found in the remaining fractures. In some areas, small particles were visible, which were contaminants after the samples (indicated by a yellow arrow). In the case of samples after 90 days of exposure, one of the fractures analyzed showed the presence of five single blastospores and one colony consisting of two cells at a distance of approximately 400 µm from the sample surface (Figure 7c,d). Blastospores or other forms of *C. albicans* were not found in the remaining failures. In some areas, only single particles were visible from which contaminants were breaking the samples.

## 4. Discussion

The research carried out thus far indicates that hard acrylic materials may be colonized and penetrated by pathogenic yeasts [21,22]. In these studies, samples of the materials were exposed to a suspension for six weeks, sectioned, and the interior of samples was examined during microscopy observations. Bulad et al. [21] found more than a half thousand blastospores at the deepest level, but a hyphae form was not observed. Krishnamurthy et al. [22] used the same hard acrylic, and an analogous sample preparation method observed blastospores and hyphae forms. These results may suggest that, in the long-term, this phenomenon may have an impact on the mechanical properties, which to a large extent determine the clinical usefulness of materials. In this study, the effect of the presence of the *C. albicans* suspension on hardness, flexural strength, tensile strength, and impact strength was investigated. The Vickers hardness test, due to the applied load and depth of the indentation marks, values should be considered as surface hardness [31,32], while the ball indentation hardness was used to check for possible changes in the macroscale. Flexural strength is considered as a fundamental property of prosthetic materials, which is particularly important when atrophy processes of the alveolar ridges lead to the creation of irregular shapes of the denture bearing area, resulting in uneven support of the prostheses [33,34]. The values of the flexural modulus affect the effectiveness of chewing [35] and the impact strength determines the resistance to dynamic loads (e.g., prosthesis fall) [7].

The conducted experiments did not show statistically significant changes in the flexural strength, flexural modulus, tensile strength, and impact strength, and the values obtained were similar to those reported in other studies for this material [36,37]. The results after exposure to the control medium confirm that changes in the basic strength properties may be insignificant in the case of the materials currently used [38], despite the fact that many previous studies have pointed to a period of even a several dozen days to a significant reduction in these properties [39,40]. The lack of changes in the properties mentioned could also be related to the use of an experimental medium other than distilled water (water is more destructive to acrylates compared to, e.g., artificial saliva) [38,41], and/or conditioning the samples in water prior to the experiment. 

Significant hardness changes have been observed after exposure to both the suspension of the control medium and *C. albicans*. In the case of the hardness measured by the ball indentation method, a decrease in hardness was recorded after the first 30 days of the experiment, which should be associated with the plasticization of the material [42,43] caused by the penetration of water molecules between the polymer chains, that weaken their interactions [44,45]. The changes measured by the ball indentation method were approximately 5% and did not translate into changes in the other mechanical properties, probably due to their relatively small influence on the changes in the yield point [46]. For the Vickers hardness, the changes were statistically significant during the 90 day experiment, but the directions of the changes were divergent; in the case of conditioning in the liquid substrate solution, an increase in hardness was recorded, while in the presence of the *C. albicans* suspension, the hardness decreased. With the applied load and the obtained hardness values, the depth of the indentations was ~25 µm, so only the hardness of the material surface was tested. Taking into account that the SEM investigations showed that the surface of the samples after exposure to the control medium showed an increasing tendency to crystallize the components of the medium with time, it can be assumed that their presence caused an increase in the hardness. In the presence of *C. albicans*, the structures formed as a result of crystallization were also visible, but a gradual decrease in the average hardness values was recorded. This process was probably related to the changes taking place in the microenvironment containing *C. albicans*. The liquid Sabouraud medium contains glucose from which *C. albicans* (like other yeasts) is capable of producing, for example, ethanol [47,48,49,50], which has a strong plasticizing effect on acrylates [44,51]. Furthermore, it has been shown that *C. albicans* in cultures containing glucose causes a significant decrease in the pH value, even from 7.5 to 3.2 in 48 h [52,53]. The reduction in the pH value in human saliva, with the addition of glucose, is caused by products of glycolysis such as acetic acid, pyruvic acid, and lactic acid [53]. A similar trend of lowering the pH value in the presence of *C. albicans* was also observed in the Sabouraud medium [54]. These data seem to be particularly important in the considered context because Miranda et al. [55] suggested that acid ingredients in liquids with lowered pH values may react with the ester groups of acrylates and lead to the creation of alcohol and carboxylic acid in materials, which further contributes to the accelerated degradation in the polymer properties. Therefore, the products generated during yeast metabolism could have contributed to changes in the properties of the experimental medium, which, in turn, caused the degradation to the surface of the PMMA samples. Due to the low intensity of the process, these changes occurred only at a slight depth in the material; therefore, the hardness test using the ball indentation method (and other mechanical properties) did not show these changes. However, the recorded decrease in hardness, although small in terms of value, should be considered as significant from a clinical point of view, because many works have emphasized the correlation of surface hardness with the tribological wear of materials [56], which is particularly important in the context of dental materials and the penetration of abrasive wear products into the body during the act of swallowing saliva.

The microscopic evaluation of the surface confirmed the presence of blastospores and the pseudohyphae of *C. albicans*, but the occurrence of hyphae was episodic. On the surfaces of the fractured samples (interior of the material), only the presence of up to a few blastospores on three fractures was observed. It can be assumed that their presence was related to accidental contamination related to the movement of the samples immediately after their destruction on the testing machine, rather than the penetration of *C. albicans* into the material. The premise in this regard may be the rare presence of other contaminations (Figure 7, yellow arrows). It should also be noted that the number of *C. albicans* cells was incomparably smaller than that reported in studies carried out in cut samples [21,22]. It should be noted that in the works cited, the sections studied were analyzed after cutting the samples in a water environment (diamond disc/microtome). However, there is no information on how (or if at all) the methodology was checked in terms of whether it was possible to transfer *C. albicans* into the material during the cutting process. In the current study, after the fracture on the testing machine, the samples were not touched in any way on the investigated surfaces, so the only possibility of contamination occurred directly after the break. Therefore, it seems that the possibility of a false positive result has been minimized to a greater extent. Another factor that could influence the results obtained was the composition of the medium in which the samples were incubated. In our study, the Sabouraud substrate was used, similar to the investigations on the colonization and penetration of *C. albicans* into prosthetic materials conducted by Burns et al. [57]. They observed the same morphological forms of *C. albicans* as in the current work, but the presence of microorganisms inside the cut samples of different types of materials was registered. In other works using a hard acrylic material [21,23], the presence of the hyphae form was confirmed, but the experimental media based on artificial saliva were used. The authors of these works do not provide the compositions of the saliva used or any of the other parameters such as the initial pH. Therefore, it is not known what the influence of these features could have been on the results of the observations, taking into account the fact that the filamentation process is favored by, for example, a pH at the level of 8, while inhibiting the hyphae growth and promoting the growth of blastospores, is favored by an acidic or neutral environment with a pH of ~4–7 such as that used in the current research or the work by Burns. However, considering that Burns et al. (based on Sabouraud medium) and the two other indicated works (based on artificial saliva) confirmed the penetration in cut samples regardless of the reported forms of *C. albicans*, it can be assumed that the medium used was not determinative for the results from the aspect of penetration. It should be noted that the works [21,22] did not discuss how the *C. albicans* cells penetrated the materials. Krishnamurthy et al. [22] linked the presence of a large number of blastospores in the interior with its potential porosity resulting from polymerization. However, such a reason seems unlikely considering that SEM studies [58,59] have proven that pores formed during crosslinking have sizes up to a few dozen nanometers, so are several dozen to one hundred times smaller than the size of blastospores, and are closed pores, so no direct migration between them is possible. At the same time, the observed tendency for the presence of *C. albicans* colonies/cells in the crystallized structures or traces after grinding is maintained, according to reports indicating that increased roughness may promote an increased degree of surface colonization [60]. This indicates that problems with the removal of microorganisms from prosthetic materials over time and their rapid recolonization after disinfection [19,61] may not be caused by the penetration of *C. albicans* into materials, but by the formation of surface microcracks or screeches due to daily use including cleaning or the impact of thermal cycles [62,63,64], which may be difficult to disinfect with cleaning agents. This supposition is supported not only by the currently obtained results, but also by the in vivo investigations by Taylor et al. [65], in which the interior of the prosthetic materials was not penetrated. These results suggest that the problem of the potential penetration of *C. albicans* into dental materials is still a challenge that requires further, intensified research.

## 5. Conclusions

The presence of the suspension of *C. albicans* did not affect the PMMA properties of the denture material such as the flexural strength, flexural modulus, tensile strength impact strength, or the ball indentation hardness. A decrease in the surface hardness was observed during the experiment, which could have been caused by the presence of yeast metabolism products, which could have caused plasticization and/or surface degradation by lowering the pH of the experimental suspension and the reactions of an acid liquid with the ester groups of acrylates. Only a few blastospores of *C. albicans* that occurred in a small part of the fractures analyzed were recorded. Considering their number and other indications resulting from the analysis of the SEM images, it should be concluded that their presence was not the result of *C. albicans* penetration into the material, but the result of accidental contamination during the mechanical properties tests. Therefore, the investigation did not confirm the penetration of *C. albicans* into the materials. The presence of blastospores and the pseudohyphae of *C. albicans* on the surface, mainly in crystallized structures and traces after grinding, indicates that under clinical conditions, deterioration of the surface quality while using dentures may be an important factor that is conducive to the colonization of materials by microorganisms. This probably led to the formation of difficult to disinfect microareas, which can be a cause of the clinically registered rapid recolonization of dentures by yeasts. The limitation was that the exposure time was not related to the actual oral conditions, but only to the specific laboratory conditions used during the study. Under clinical conditions, there are many additional factors that could influence the results, and these factors should be considered in future tests. The creation of biofilm may be especially important in this context. In the presented work, as in our previous experiments in the field, a biofilm was not formed despite a long exposure time, which may be related to the use of a diluted substrate, so in the future, similar experiments with biofilm formation should be carried out.

## Figures and Tables

**Table 1:** The results of the Tukey HSD post hoc tests (columns) and the Student *t*-tests (rows) for the ball hardness.

Exposure Time	Control Medium	*C. albicans*
24 h H_2_O	A	A
30 days	B; a	B; a
60 days	B; a	B; a
90 days	B; a	B; a

The same uppercase letters (A–B) for each column and the lowercase letters for each row did not show significantly different results at the level of *p* < 0.05.

**Table 2:** The results of the Tukey’s HSD post hoc tests (columns) and *t*-student tests (rows) for the Vickers hardness.

Exposure Time	Control Medium	*C. albicans*
24 h H_2_O	A	A
30 days	A; a	A,B; a
60 days	A; a	B,C; b
90 days	B; a	C; b

The same uppercase letters (A–C) for each column and the lowercase letters (a–c) for each row did not show significantly different results at the level of *p* < 0.05.

## Data Availability

Data supporting reported results are available from the authors.
